# Bioactive Natural and Synthetic Products in Human Health and Diseases: Basic, Preclinical and Clinical Studies

**DOI:** 10.3390/nu16193265

**Published:** 2024-09-27

**Authors:** Felicite K. Noubissi, Tolulope O. Omolekan, Anthony L. Walker, Jean Christopher Chamcheu

**Affiliations:** 1 Department of Biology, Jackson State University, Jackson, MS 39217, USA; 2 Department of Biological Sciences and Chemistry, College of Sciences and Engineering, Southern University and A&M College, Baton Rouge, LA 70813, USA; 3 Department of Pathological Sciences, School of Veterinary Medicine, Louisiana State University, Baton Rouge, LA 70803, USA; 4 Department of Biochemistry, Bowen University Iwo, 232101, Osun State, Nigeria; 5 School of Clinical Sciences, College of Pharmacy, University of Louisiana, Monroe, LA 71209, USA; 6 Department of Pathology and Translational Pathobiology, Louisiana State University Health Sciences Center, Shreveport, LA 71103, USA

## 1. Introduction

Since the early 20th century, the increase in non-communicable diseases such as diabetes, heart disease, stroke, and cancer compared to infectious diseases has led to chronic illnesses becoming a leading cause of global morbidity and mortality [1]. Despite the medical advances observed in the treatment of various ailments, the incidence and prevalence of chronic diseases such as diabetes, stroke, and cancer continue to escalate, resulting in life-threatening, costly, and debilitating consequences that reduce life expectancy. Additionally, chemotherapy often leads to drug resistance and reduced effectiveness, underscoring the urgent need for more efficient prevention and treatment methods [1]. Annually, chronic diseases account for 41 million deaths globally, constituting 71% of all fatalities [1]. Of these deaths, over 15 million occur in individuals aged 30–69, with 85% of premature deaths occurring in low- and middle-income countries. The global cost of chronic diseases is projected to reach USD 47 trillion by 2030 [1]. Lifestyle choices and community factors significantly influence the onset and management of chronic diseases. Many of these conditions could be prevented by addressing risk factors such as smoking, alcohol consumption, obesity, a poor diet, and a lack of physical activity. Unfortunately, investments in prevention are minimal compared to those in treatment, both in terms of lifestyle changes and the broader social determinants of health. Considering the projected increase in chronic disease, advancements in technology, nutraceuticals, and pharmaceuticals, coupled with increased preventive measures, are crucial.

Plant-based bioactive products that are abundantly found in fruits and vegetables, alongside their synthetic equivalents, are gaining recognition for the roles they play in promoting health, preventing diseases, and providing therapeutic benefits in both modern and traditional medicine. In recent decades, the rigorous testing of these products using appropriate in vitro and preclinical models has led to the discovery of phytochemicals, plant-derived pharmaceuticals, and other formulations collectively known as “nutraceuticals”, offering insights into their health benefits [2,3,4]. These products are prized or valued for their nutritional and medicinal properties, significantly enhancing individuals’ well-being, immune function, longevity, quality of life, and participation in activity [5]. Many contemporary drugs, inspired by traditional remedies, originate from these natural sources and contribute to the improvement of health through dietary intake, ingredients, or supplements; this is often due to their antioxidative, anticancer, antimicrobial, immunomodulatory, and antihypertensive effects [6,7]. In the management of chronic conditions and drug-resistant infections, nutraceuticals complement conventional medicine by addressing the causes of disease and enhancing the body’s healing mechanisms [6,8,9]. They are also believed to promote longevity by offering protection against aging, environmental threats, oxidative stress, infections, and chronic inflammatory conditions such as acne, atopic dermatitis, cardiovascular diseases, hypertension, psoriasis, diabetes, diabetic ulcers, chronic wounds, various cancers, obesity, and related risk factors [7,10,11].

This Special Issue aims to offer an overview of current research on natural dietary compounds, pharmaceuticals, extracts, and other preparations, focusing on their roles in human health and diseases. It highlights the transition from traditional medicine and anecdotal evidence to an evidence-based approach to preventing or managing chronic diseases that significantly impacts human health and wellness. This Special Issue comprises 23 contributions, including global studies on bioactive products and their synthetic equivalents, human trials, animal models, in vitro studies, and cell line research conducted both in the laboratory and in real-world settings. Among these contributions, 21 are original research articles and 2 are review articles. The findings highlight their bioavailability and involvement in physiological processes, molecular targets and the regulation of pathways, and the relevant mechanisms of action.

## 2. An Overview of Published Articles

Several groups (Christiansen, Wan, Kang, Baik, Rebello, Koonyosying, Anbualakan and their colleagues) investigated the health-promoting and therapeutic effects of beverages, legumes, vegetables, and fortified products on chronic diseases such as diabetes, depression, hyperlipidemia, hypertension, and obesity. Their research involved randomized clinical trials that explored the physiological impacts of nutraceutical-containing oral formulations and fortified foods on healthy individuals and patients. Exploring the high antioxidant contents of *Aronia melanocarpa* berries (AMB) and their potential ability to treat type 2 diabetes mellitus (T2DM), Christiansen et al. (contribution 1) investigated the effects of fermented and non-fermented berry pulp in T2DM patients compared to wheat bran (placebo). Their findings revealed that neither fermented nor non-fermented berry extracts significantly affected hemoglobin A1c, blood sugar (fructosamine, glucose) levels, insulin, or glucagon-like peptide-1. However, participants who consumed fermented AMB extract exhibited increased glucose-dependent insulinotropic peptide (GIP) levels in fasting blood samples compared to those who received the placebo. Meanwhile, the overall clinical significance of these extracts remains uncertain and thus requires further studies.

Considering the array of bioactive compounds in green tea, Wan et al. (contribution 2) suggested its consumption as an effective strategy for reducing the risk of depression. They explored its impact on hormonal fluctuations, a significant factor in depression among postmenopausal women that are typically managed with antidepressants and lifestyle changes. While investigating the impact of long-term green tea consumption on postmenopausal women from a tea-producing village, they found substantial reductions in insomnia, depression, the basal metabolic index and the systemic immune inflammation index levels in the tea-drinking group compared to the control, as well as an increase in estradiol. These findings suggest that, by reducing inflammation and enhancing estradiol levels, green tea consumption may reduce the risk of depression, highlighting it as a promising lifestyle intervention. Additionally, the extended literature review by Anbualakan et al. (contribution 3) summarizes the ability of the flavonoids and carotenoids found in tea and various fruits such as blueberries, lemons, carrots, tomatoes, and grapes to act as protective compounds against skin photodamage. They underscored the listed food items as rich sources of phytochemicals (flavonoids and carotenoids) that could prevent UV-induced skin damage. This indicates that these phytochemicals could be used clinically and cosmetically to prevent sunburn, photoaging and UV-related skin cancers, as well as delay premature aging and skin cancer.

Prolonged postprandial hyperlipidemia is a risk factor for atherosclerosis and endothelial dysfunction, which are key factors in the development and progression of cardiovascular disease [12,13]. It is closely associated with coronary heart disease and involves oxidative stress and inflammation [13]. Bioactive compounds that regulate glucose metabolism, possess intrinsic antioxidant activity, and modulate inflammation can enhance lipid metabolism, reduce postprandial hyperlipidemia, and lower the risk of cardiovascular diseases. In this context, Kang et al. (contribution 4) evaluated the non-fasting triglyceride levels, which are considered more precise indicators of cardiovascular risk than fasting levels, in adults consuming a high-fat diet with low amounts of fruits and vegetables after consuming *Platycodi radix* beverage (PR). Their findings indicated that PR consumption significantly alters adults’ blood microbiota profiles and improves the clearance of triglyceride-rich lipoproteins after meals with such dietary patterns, suggesting its potential benefits for cardiovascular health.

In their study, Baik and colleagues (contribution 5) showed that aged or fermented garlic extract (FGE) acts as a natural remedy with the ability to enhance vascular function. This enhancement occurs through the increased bioavailability of vascular nitric oxide (NO), which is facilitated by the bioconversion of nitrite to NO and its subsequent conversion back to nitrite (NO^2−^) by microorganisms during fermentation. Significant changes in blood pressure (BP) and the velocity of the common carotid artery (CCA) were observed within 30–60 min post-FGE consumption in healthy adults. Additionally, FGE intake was associated with an increase in regional cerebral blood flow (rCBF) and body surface temperature, attributed to changes in peripheral blood flow (PBF). No clinical side effects were detected. Therefore, the oral administration of FGE, which contains NO^2−^, enhances vascular function, including CCA, BP, rCBF, and PBF.

Rebello et al. (contribution 6) demonstrated that soybean products, including whole green soybean pods processed into flour, are safe and tolerable. This is due to their nutrient, dietary fiber, and phytoalexin contents, which benefit cardiovascular and overall health. They found that adults with obesity tolerated 30 g of soybean flour well, and that it also induced a feeling of fullness. Interestingly, processed soybean flour (PSF) has a reduced content of iron and oligosaccharides, which potentially reduces flatulence. The study suggests that lower doses of soybean flour could be beneficial for overall health and may help prevent obesity in older adults.

In their study, Koonyosying et al. (contribution 7) evaluated the health impacts and sensory acceptability of perilla fruit oil (PFO)-fortified soybean milk (PFO-SM) in a randomized clinical trial with healthy participants. They observed that PFO-SM consumption contributed to a reduction in the levels of serum triglycerides and erythrocyte reactive oxygen species, and an increase in phagocytosis and serum antioxidant activity. They also found that soybean milk fortified with 1% PFO was the most accepted formulation in their study. These findings suggest that PFO fortification in soybean milk may bolster or enhance radical-scavenging and phagocytotic functions, potentially reducing the risk of chronic diseases.

Other studies (Kapoor, Kang, Kozłowski and colleagues) utilized randomized clinical trials to investigate the pharmacokinetic and therapeutic effects of bioactive products and synthetic analogs in human participants. Kapoor et al. (contribution 8) demonstrated the effects that a soluble complex of hesperetine-7-*O*-glucoside with β-cyclodextrin (HEPT7G/βCD) (which is a synthetic derivative of hesperetin found in citrus) has on vasodilation, endothelial function, and cold-induced stress in healthy individuals. Their study showed a significant increase in skin blood flow after consuming small doses of HEPT7G/βCD, effectively restoring peripheral skin temperature. However, they noted variations in the time taken for this increase in blood flow to appear with different doses of the HEPT7G/βCD complex, suggesting that hesperetin metabolites, once deconjugated, may specifically modulate skin blood-flow-dependent thermoregulation in humans.

Kang et al. (contribution 9) conducted a comparative study on the pharmacokinetics of chitosan-derived and biofermentation-derived glucosamine, both used as nutritional supplements for osteoarthritis in humans. Their study concluded that while both types of glucosamine met or satisfied the bioequivalence standards, they differed in their mean peak plasma concentration (Cmax) ratios. However, the sequence, period, or origin of glucosamine did not significantly impact the pharmacokinetic parameters. These results suggest that biofermentation-derived glucosamine could serve as a sustainable alternative for glucosamine supplementation.

To unravel the complex factors influencing the sebaceous–hair unit and the development of acne, Kozłowski et al. (contribution 10) compared metabolic parameters and the severity of acne before and after three types of treatments in 168 women: contraceptive preparations alone, contraceptive preparations with cyproterone acetate, and contraceptive preparations with isotretinoin. They assessed the correlation between the metabolic parameters, the severity of the women’s acne, and the impact of treatment on the women’s intake of dairy or sweets. The study found that the women’s low-density lipoprotein levels and consumption of sweets correlated with the severity of their acne. While contraceptives containing ethinylestradiol and drospirenone-based contraceptives remain fundamental in acne treatment, no significant link was observed between the severity of acne and the consumption of dairy or sugar before and after the treatment.

Thirteen research teams employed in vitro cell cultures and in vivo preclinical murine models to investigate the efficacy and pharmacokinetics of bioactive products and their derivatives for health enhancement. They explored natural products, phytochemicals, dietary lipids, and hormones in studies on hair regeneration, cancer, atopic dermatitis, testicular and kidney damage, ulcers, and obesity. Wang et al. (contribution 11) demonstrated that hordenine, which is found in various plants such as cacti and germinated barley seeds, exhibits anti-inflammatory properties, can inhibit hyperpigmentation, and combats diabetes, fibrosis, and lung cancer. Their research demonstrated that hordenine stimulates the proliferation and activity of dermal papilla cells, thereby promoting hair regeneration in mice via the activation of the Wnt/β-catenin signaling pathway with minimal toxicity. These findings suggest that hordenine could be a promising candidate for the development of drugs targeting the prevention or treatment of alopecia.

In a novel study, Mohamad Ali et al. (contribution 12) investigated the impact of the lipid carrier structure on the ability of Docosahexaenoic acid (DHA) consumption to lower the risk of cancer; they evaluated the cytotoxicity of different DHA lipid carrier formulations on the MDA-MB-231 breast cancer cell line. They demonstrated that glycerophinphatidylcholine-based DHA carriers significantly reduced cell viability, with 1-docosahexaenoyl-2-hydroxy-sn-glycero-3-phosphocholine (LPC-DHA) being the most effective formulation. The research suggests that LPC-DHA exerts cytotoxic effects by inducing oxidative stress and membrane damage in MDA-MB-231 cells, as evidenced by increased levels of heme oxygenase 1 (HO-1), superoxide dismutase 2 (SOD-2), and lactase dehydrogenase (LDH) activation.

Similarly, Adinew et al. (contribution 13) demonstrated that thymoquinone (TQ), a compound obtained from black cumin (*Nigella sativa* L.) seed, induces cytotoxicity, inhibits clonogenicity, reduces migration and invasion, and promotes cell cycle arrest in two genetically different triple-negative breast cancer (TNBC) cell lines (MDA-MB-231, MDA-MB-468), mostly at the S phase. They also showed that TQ induces apoptosis in these cell lines by upregulating different sets of apoptotic genes. Their findings indicated that the MD-MB-468 cell line responds more favorably to TQ than the MDA-MB-231 cell. Therefore, TQ might serve as a potential drug for the treatment of chemo-resistant TNBC.

Escudero-Feliu et al. (contribution 14) investigated the effects of three β-conglutin isoforms obtained from narrow-leafed lupin (*Lupinus angustifolius* L.) on three breast cancer cell lines: MDA-MB-231 (high levels of p53 mutant with gained functionality, TNBC), MCF-7 (p53 wild-type, ERα positive, and weak for HER2), and SK-BR-3 (p53 mutant without gained functionality, ERα negative, and HER2-positive). They demonstrated that β1, β3, and β6-conglutin isoforms can inhibit the growth of these breast cancer cells. The isoforms exerted cytotoxic effects on the cells through autophagy or ferroptosis, depending on the cell line and p53 status. Moreover, they observed a reduction in the stemness characteristics of aggressive breast cancer cells, including the Luminal B and TNBC subtypes, which could potentially serve as sensitizers for chemotherapy.

Bergandi et al. (contribution 15) found that whey-derived products and their purified galactooligosaccharides (GOS) enhance wound healing and skin health by improving the functions of keratinocyte and acting as toll-like receptor ligands. These products triggered nuclear factor kappa B (NF-kB) signaling, increased the levels of interleukin-8, and promoted cell migration and the reduction of E-cadherin without causing epithelial–mesenchymal transition, thereby accelerating wound healing. Furthermore, the functionality of mitochondria in keratinocytes treated with GOS improved as the Forkhead Box O1 (FOXO-1) transcription factor was expelled from the mitochondria following GOS treatment, thereby alleviating the repressive activity of FOXO-1 in the mitochondria. The fermented product made by native microorganisms was particularly effective in altering the activity of keratinocyte, underscoring the health benefits of whey derivatives.

In 2022, two reports by Arunachalam et al. (contribution 16) and AlAsmari et al. (contribution 17) showed that phytochemicals such as α-bisabolol and geraniol could mitigate the testicular and kidney damage associated with Doxorubicin (DOX), a chemotherapeutic agent widely used in cancer treatment that has limited efficacy due to its toxicity. Arunachalam et al. (contribution 16) indicated that in a rat model of DOX-induced testicular injury, α-bisabolol attenuated the activation of NF-κB/MAPK signaling and endoplasmic reticulum-stress-mediated apoptosis by activating Nrf2-mediated antioxidant defenses, thereby shielding the testes from DOX-induced toxicity; this includes weight loss, oxidative stress, inflammation, and ER-stress-mediated testicular apoptosis. Their findings suggest that α-bisabolol could serve as a protective agent or adjuvant to chemotherapeutic drugs, potentially reducing their detrimental effects on organs during chemotherapy, enhancing the drugs’ effectiveness and reducing morbidity in cancer patients. Similarly, AlAsmari et al. (contribution 17) showed that administering geraniol to rats prior to an injection of DOX mitigated the DOX-induced alterations in the kidneys’ antioxidant parameters, enzymatic activities, and the expression of genes and proteins related to inflammation and apoptosis. Furthermore, this pre-treatment with geraniol maintained the histological integrity of the kidneys in a dose-dependent manner, indicating its potential ability to safeguard against renal dysfunction caused by DOX.

Additionally, Kim et al. (contribution 18) and Woo et al. (contribution 19) evaluated the therapeutic potential application of Korean Red Ginseng (*Panax ginseng* C.A. Mey.) extract to both Helicobacter pylori-infected gastric epithelial AGS cells and an atopic dermatitis (AD) murine model. Kim et al. (contribution 18) discovered that the extract alleviated inflammation and mitochondrial dysfunction in *H. pylori*-infected AGS cells by disrupting Nrf2–Keap1 interactions, thereby facilitating the nuclear translocation of Nrf2. This activation of Nrf2 led to the induction of superoxide dismutase 1 (SOD-1) and heme oxygenase 1 (HO-1), leading to a reduction in the expression levels of IL-8 and ROS. Moreover, their findings indicated that Korean Red Ginseng extract partially restored mitochondrial function by enhancing the mitochondrial membrane potential and ATP levels, which are typically compromised during *H. pylori* infection. In a related study, Woo et al. (contribution 19) demonstrated that Korean Ginseng extract, when used alongside conventional systemic therapies, significantly lessened the severity of AD, which is a chronic cutaneous disease. This combination therapy diminished dermal inflammation, lowered the levels of immunoglobulin E, and decreased the expression of CD1a and IL-17, indicating that Korean Red Ginseng, alongside standard treatments, can effectively manage AD.

Meanwhile, in pursuit of sustainable methods for the production of vegetative biomass and phytochemicals with therapeutic potential, Povydytsh et al. (contribution 20) demonstrated that biomass from *Dioscorea deltoidea*, *Tribulus terrestris*, and *Panax japonicus* cultivated in bioreactors retained their therapeutic effectiveness. Evidentially, these plants demonstrated their ability to reduce weight gain and the fat mass proportion in obese rats fed a high-fat diet. Additionally, infusions derived from these plants mitigated intracellular dehydration, balanced intra- and extracellular fluids, and notably decreased the blood glucose and cholesterol levels in rats. Among the three, *D. deltoidea* was particularly effective in reducing body fat mass and restoring the balance of cellular fluids without adversely affecting reproductive functions.

In addition, Lupo et al. (contribution 21) reported the development of a novel oral formulation of berberine using Sucrosomial^®^ technology, a patented system designed to enhance the absorption and efficacy of minerals and micronutrients. This formulation aimed to improve gastrointestinal absorption, reduce insulin resistance, and evaluate its pharmacokinetics and effectiveness in Huh7 cell lines and in mice fed both standard and high-fat diets. The in vitro model demonstrated that the formulation did not compromise the cell viability of Huh7 up to concentrations of 40 µM. Moreover, it activated glucokinase (GK) and phosphorylated 5′-adenosine monophosphate (AMP)-activated protein kinase (AMPK), which are both crucial in managing insulin resistance. In mice, the formulation resulted in an eightfold increase in plasma concentration after three weeks of oral administration compared to standard berberine. It also significantly enhanced the accumulation of reduced, demethylated, and glucuronide forms of berberine in the brain. This formulation represents an innovative and effective approach to enhancing the gastrointestinal absorption of berberine. Additionally, Laurindo et al. (contribution 22) conducted a review of 3418 studies on the therapeutic effects of pomegranate on the risk factors for metabolic syndrome (MetS). Their findings suggest that pomegranate may aid in reducing body weight, triglycerides, and total cholesterol, as well as increase high-density lipoprotein cholesterol levels, normalize blood pressure and glycemia, and improve insulin resistance. Nonetheless, further well-designed clinical trials should be performed in order to establish appropriate formulations and dosages for the prevention or treatment of MetS.

Moreover, Habib et al. (contribution 23) demonstrated that the extract of date palm fruit seed (*Phoenix dactylifera* L.), known as DSE, is a rich source of polyphenols with significant antioxidative and disease-preventing properties. Their research revealed that DSE can inhibit iron activity, DNA damage, and the activities of acetylcholinesterase and tyrosinase. Additionally, DSE was found to reduce the proliferation of cancer cells by inducing apoptosis, downregulating Bcl-2 and p21 genes, and upregulating p53 expression in both untreated and 5-FU treated cells. These findings indicate the potential use of DSE in the development of functional foods and as a natural chemotherapeutic agent.

## 3. Conclusions

Overall, the 23 papers published in this Special Issue highlight the pharmacokinetics, bioavailability, and role that natural bioactive compounds and synthetic products play in physiological processes; this Special Issue also highlights the impact of molecular targets, pathways, and their mechanisms of action on human health and diseases through basic, preclinical and clinical studies. In conclusion, the regular consumption of natural products such as fruits, beverages, and vegetables, alongside their synthetic analogs, presents a cost-effective and healthy approach to improving human health. This practice significantly reduces the risks associated with chronic diseases and enhances overall health, underscoring the importance of using nutraceuticals as a stand-alone, adjuvant, or combination strategy for the prevention and management of various chronic diseases.

Natural dietary products are potential sources of therapeutic small molecules and their synthetic derivatives, which are important for the development of new drugs. A range of products have shown promising health-promoting effects due to their anti-aging and anti-oxidative, anti-inflammatory, chemopreventive, and chemotherapeutic properties. Nonetheless, further research using physiologically relevant models and large population studies is essential in order to determine their efficacy and potential clinical applications.
